# High-resolution genomic and expression analyses of copy number alterations in HER2-amplified breast cancer

**DOI:** 10.1186/bcr2568

**Published:** 2010-05-06

**Authors:** Johan Staaf, Göran Jönsson, Markus Ringnér, Johan Vallon-Christersson, Dorthe Grabau, Adalgeir Arason, Haukur Gunnarsson, Bjarni A Agnarsson, Per-Olof Malmström, Oskar Th Johannsson, Niklas Loman, Rosa B Barkardottir, Åke Borg

**Affiliations:** 1 Department of Oncology, Clinical Sciences, Lund University, Barngatan 2B, SE 22185 Lund, Sweden; 2 CREATE Health Strategic Center for Translational Cancer Research, Lund University, BMC C13, SE 22184, Lund, Sweden; 3 Department of Pathology, Clinical Sciences, Lund University, University Hospital, SE 22185 Lund, Sweden; 4 Department of Pathology, Landspitali-University Hospital, 101 Reykjavik, Iceland; 5 Faculty of Medicine, University of Iceland, 101 Reykjavik, Iceland; 6 Department of Oncology, Landspitali-University Hospital, 101 Reykjavik, Iceland; 7 Lund Strategic Research Center for Stem Cell Biology and Cell Therapy, Lund University, BMC B10, SE 22184, Lund, Sweden

## Abstract

**Introduction:**

*HER2 *gene amplification and protein overexpression (HER2+) define a clinically challenging subgroup of breast cancer with variable prognosis and response to therapy. Although gene expression profiling has identified an ERBB2 molecular subtype of breast cancer, it is clear that HER2+ tumors reside in all molecular subtypes and represent a genomically and biologically heterogeneous group, needed to be further characterized in large sample sets.

**Methods:**

Genome-wide DNA copy number profiling, using bacterial artificial chromosome (BAC) array comparative genomic hybridization (aCGH), and global gene expression profiling were performed on 200 and 87 HER2+ tumors, respectively. Genomic Identification of Significant Targets in Cancer (GISTIC) was used to identify significant copy number alterations (CNAs) in HER2+ tumors, which were related to a set of 554 non-*HER2 *amplified (HER2-) breast tumors. High-resolution oligonucleotide aCGH was used to delineate the 17q12-q21 region in high detail.

**Results:**

The *HER2*-amplicon was narrowed to an 85.92 kbp region including the *TCAP*, *PNMT, PERLD1, HER2, C17orf37 *and *GRB7 *genes, and higher *HER2 *copy numbers indicated worse prognosis. In 31% of HER2+ tumors the amplicon extended to *TOP2A*, defining a subgroup of HER2+ breast cancer associated with estrogen receptor-positive status and with a trend of better survival than HER2+ breast cancers with deleted (18%) or neutral *TOP2A *(51%). HER2+ tumors were clearly distinguished from HER2- tumors by the presence of recurrent high-level amplifications and firestorm patterns on chromosome 17q. While there was no significant difference between HER2+ and HER2- tumors regarding the incidence of other recurrent high-level amplifications, differences in the co-amplification pattern were observed, as shown by the almost mutually exclusive occurrence of 8p12, 11q13 and 20q13 amplification in HER2+ tumors. GISTIC analysis identified 117 significant CNAs across all autosomes. Supervised analyses revealed: (1) significant CNAs separating HER2+ tumors stratified by clinical variables, and (2) CNAs separating HER2+ from HER2- tumors.

**Conclusions:**

We have performed a comprehensive survey of CNAs in HER2+ breast tumors, pinpointing significant genomic alterations including both known and potentially novel therapeutic targets. Our analysis sheds further light on the genomically complex and heterogeneous nature of HER2+ tumors in relation to other subgroups of breast cancer.

## Introduction

Gene amplification is a frequent mechanism of oncogene activation in breast cancer (BC) [1]. Amplification and overexpression of the *HER2 *(HER2/neu, *ERBB2*) oncogene on chromosome 17q12 occur in 15-25% of invasive BC [2]. *HER2*-amplified (HER2+) tumors define a clinically important BC subgroup, generally associated with poor prognosis [2,3]. Strategies to therapeutically target the HER2 protein by monoclonal antibodies (for example, trastuzumab) or tyrosine kinase inhibitors (for example, lapatinib) have been successful [4-7]. As these drugs are most effective in HER2+ BC, considerable efforts have been devoted to accurate assessment of HER2 status, currently performed by immunohistochemistry (IHC) and/or in situ hybridization [8]. However, despite the success of targeted treatment, many HER2+ cases fail to respond or develop resistance over time.

It is evident that the *HER2*-amplicon has a variable structure, comprising other genes in the 17q12-q21 region that may contribute to tumor progression and treatment effect in HER2+ BC. One of these genes is topoisomerase IIα (*TOP2A*), located 700 kb telomeric of *HER2*, that may be either co-amplified, unaffected or deleted in HER2+ tumors [9]. TOP2A status has been reported to significantly influence the response to anthracycline-based therapy [10-12] although conflicting results exist [13,14]. Furthermore, it is evident that HER2+ tumors constitute a biologically heterogeneous subgroup of BC. Global gene expression profiling defines an ERBB2 molecular subtype of BC that predominantly consists of estrogen receptor (ER) negative HER2+ tumors (HER2+/ER-) [15,16], while HER2+/ER+ tumors are more heterogeneously classified. In addition, we recently used gene expression profiling to characterize three distinct subgroups of HER2+ tumors, and to create a HER2-derived prognostic gene signature with strong correlation to outcome for patients with HER2+ disease [17].

Genomic profiling using array comparative genomic hybridization (aCGH) analysis has revealed frequent complex copy number alterations (CNAs) on chromosome 17q, often including high-level amplifications, in HER2+ BC [18-20]. Moreover, although HER2+ tumors share other commonly gained or lost regions with non-*HER2 *amplified (HER2-) tumors, the genomic profiles of HER2+ tumors are more often heterogeneous and complex in nature [18-20]. We designed a study to comprehensively investigate CNA patterns in 200 HER2+ tumors using high-density bacterial artificial chromosome (BAC) aCGH in concert with custom-designed high-density zoom-in aCGH [21]. We provide evidence of considerable genomic heterogeneity in HER2+ BC, and further delineate the boundaries of the 17q12-q21 amplicon. In addition, matched global gene expression profiles were available for 87 tumors allowing correlation of CNAs to mRNA expression levels for identification of putative target genes.

## Materials and methods

### Patients and tumor material

Freshly frozen HER2+ BC tissue (n = 188) was obtained from the Southern Sweden Breast Cancer Group's tissue bank at the Department of Oncology, Lund University Hospital and from Department of Pathology, Reykjavik University Hospital. Additionally, 12 formalin-fixed paraffin embedded (FFPE) tumors were obtained from the Department of Pathology, Lund University Hospital. Confirmatory IHC and/or fluorescence *in situ *hybridization (FISH) data were available in 69 of 200 HER2+ tumors [17,22]. Patient and tumor characteristics are summarized in Table 1 and described in detail in Additional file 1. The study was approved by the regional Ethical Committee in Lund (reg. no. LU240-01 and 2009/658), waiving the requirement for informed consent for the study, and the Icelandic Data Protection Committee and the National Bioethics Committee of Iceland. For Icelandic patients written informed consent was obtained according to the national guidelines.

**Table 1:** Patient and tumor characteristics for the 200 HER2+ tumors and the 554 HER2- reference breast cancer data set

	HER2+	HER2-*
Number of tumors	200	554
Number of primary tumors	176	--
Number of metastases	5	--
Unknown status	19	--
**Tumor size**		
≤20 mm	57	205
>20 mm	134	226
Mean size mm (SD)	29 (16)	26 (14)
**Histological grade**		
Grade 1	1	41
Grade 2	26	134
Grade 3	38	134
**Estrogen receptor status**		
Positive	76	306
Negative	122	149
**Lymph node status**		
Negative	69	244
Positive	123	194
**Age**		
Median age in years (range)	56 (27 to 84)	55 (28 to 94)
<50 years	77	212
≥50 years	109	252
**DNA ploidy status**		
Aneuploid	112	--
Diploid	39	--
Unknown	49	--
Gene expression subtype**		
Basal-like	7	135
ERBB2	51	6
Normal-like	9	35
Luminal A	4	153
Luminal B	10	77
Unclassified	6	42
Overall survival***		
Number of deaths	109	190
Within five years	80	107
Median survival in years (range)	7 (0.16 to 18.5)	7.6 (0.1 to 31.9)
Median follow-up in years for patients still alive (range)	12.8 (7 to 18.5)	10.2 (1.5 to 20.2)

*: The HER2- data set is composed of four individual data sets as described in **Material and methods**.

**: Classification in gene expression subtypes according to Hu et al. [41].

***: For primary HER2+ tumors only.

### aCGH analysis

BAC microarrays were produced by the SCIBLU Genomics Center [23] in three array formats, 32K (Gene expression Omnibus, GEO, GPL4723), 33K (GPL7247) and 38K (GPL9077) all mapped to the UCSC Human Genome browser build 17 [24]. DNA from fresh frozen and FFPE tumor tissue was extracted as described (Additional file 2). Array printing, labeling, hybridization, scanning and image analysis were performed as previously described [25]. Technical replicate experiments were performed for 15 tumors. Copy number estimates (log_2_ratios) for each array were normalized [26] and replicated samples were merged after normalization. Breakpoint analysis was performed using circular binary segmentation (CBS) with α = 0.01 [27]. Only segments ≥4 BAC probes were used in further analyses. Following segmentation, array platforms were combined into a common array design. CNAs were detected using sample adaptive thresholds from 250 kb smoothed data [26] (Additional file 1). Threshold for amplification was set to segmented log_2_ratio ≥0.5, and for high-level amplification to segmented log_2_ratio ≥1 for HER2+ tumors. Recurrent high-level amplifications were defined as single peaks computed from the shortest region of amplification overlap occurring in >2% of tumors. *HER2/TOP2A *co-amplification was defined as segmented log_2_ratio ≥0.5 for *HER2 *and *TOP2A*. Co-amplification percentages were calculated as the number of tumors with co-amplification divided by the lowest number of the individual amplifications, if not stated otherwise. Pericentromeric BAC probes on the p- and q-arm of chromosome 17 were identified as the three probes closest to the chromosome 17 centromer (CEP17) (Additional file 2). CEP17 amplification was defined as the average segmented log_2_ratio ≥0.5 of either the probes on the p- or q-arm. BAC aCGH data are available through GEO [28] as [GEO:GSE21259].

### Zoom-in aCGH analysis

Custom-designed 60-mer oligonucleotide zoom-in aCGH arrays with an average probe-to-probe spacing of 100 bp in the 17q12-q21 region were designed using the Agilent eArray ver. 5.3 software (Agilent Technologies, Santa Clara, CA) as described (Additional file 2) and performed on 20 tumors (Additional file 1). Microarrays were processed as described [21]. Breakpoint analysis was performed using CBS (α = 0.01). Agilent probes were mapped to the UCSC build 18 [24]. Thresholds for amplification and high-level amplification were set similarly as for BAC aCGH data.

### Identification of significant copy number alterations and fraction of the genome altered

Genomic Identification of Significant Targets in Cancer (GISTIC) [29] was used to identify significant CNAs in the 200 tumors (Additional file 2). GISTIC regions with q-value < 0.25 were identified as significant. Student's t-test performed on average log_2_ratios for GISTIC regions were used to identify regions associated with different clinical variables, such as ER status, lymph node (LN) status, histological grade (grade 3 vs. 1 and 2), DNA ploidy (diploid vs. aneuploid), tumor size (≤20 mm vs. >20 mm) and patient age (<50 years vs. ≥50 years) for HER2+ tumors. A false discovery rate-adjusted *P*-value < 0.05 was considered significant (Additional file 2). Genomic coordinates for GISTIC regions are mapped to the UCSC Human Genome browser build 17 [24]. A firestorm-like amplification pattern [30] was defined as at least three high-level amplifications larger than three BAC probes located on the same chromosomal arm, separated by non-amplified segments, and with a maximum inter-peak distance <50% of the chromosome arm length. The fraction of the genome altered (FGA) was calculated as previously described [17].

### External aCGH data sets for comparison

CNAs and amplification frequencies in HER2+ tumors were compared to an assembled HER2- reference BC data set (n = 554) comprising four BC aCGH data sets, Chin *et al. *[31], Fridlyand *et al. *[32], Adelaide *et al. *[33], and Jönsson *et al. *(submitted) (Table 1 and Additional file 3). Data sets were processed individually (Additional file 2), transformed to a common 100 kb probe set as described [34], and merged. Clinical follow-up information was available for the Chin, Fridlyand, and Jönsson data sets. Gene expression subtype classification [15] was available for the Chin, Adelaide, and Jönsson data sets (Additional file 2). Threshold for amplification was set to segmented log_2_ratio ≥0.5, and for high-level amplification to segmented log_2_ratio >0.8 for HER2- tumors in the reference data set.

### Gene expression analysis

Gene expression profiles for 87 HER2+ tumors were available as either oligonucleotide data (n = 58, Jönsson *et al. *submitted) or cDNA data (n = 29) [22] part of larger BC data sets. Data sets were individually processed and classified according to different gene signatures (Additional file 2).

### Correlation of gene expression data with genomic aberrations

Gene expression data were compared to GISTIC aCGH log_2_ratios using Pearson correlation as described [25]. A correlation cut-off representing *P *= 0.05 obtained from 10,000 permutations of aCGH sample labels was used to identify significantly correlated genes in GISTIC regions. Global correlation analysis using genes mapped to individual BAC probes was performed similarly, with two modifications; segmented log_2_ratios were used for individual BAC probes and 1,000 permutations were performed for *P*-value estimation.

### Survival analysis

Overall survival (OS), univariate and multivariate regression analyses were performed in R [35] using the Survival package. Survival curves were compared using Kaplan-Meier estimates and the log-rank test. The full follow-up time was used for log-rank tests and regression analyses if not specified otherwise. In multivariate analysis stratified tumor size and LN status were included as covariates. Tick marks in Kaplan-Meier plots indicate censored follow-up.

## Results

### Extent and patterns of 17q12-q21 amplification in HER2+ breast cancer

**Figure 1:**
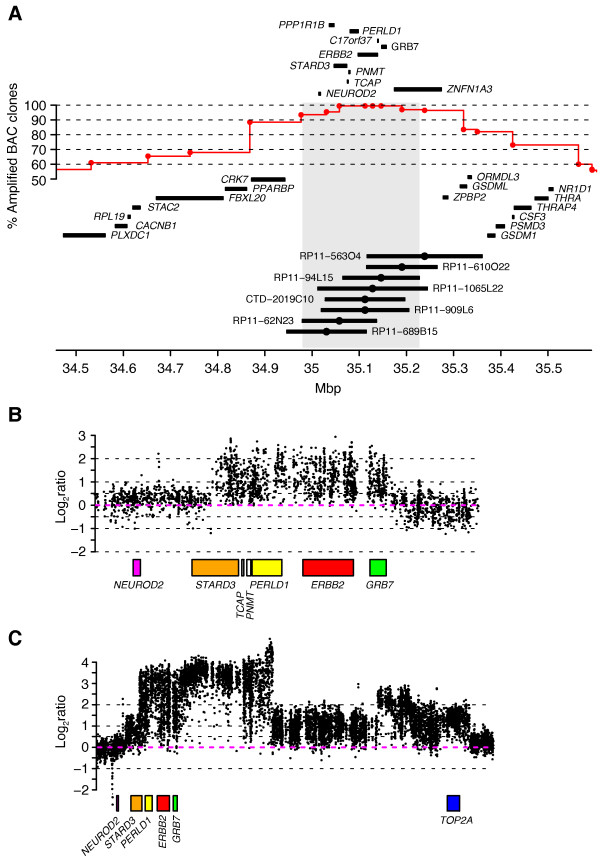
**Extent and pattern of the *HER2*-amplicon on chromosome 17q12-q21 in HER2+ BC**. (**A) **Frequency of amplification across 200 *HER2*-amplified tumors analyzed by BAC aCGH. Frequency estimates correspond to number of tumors with segmented log_2_ratio >0.5 for respective BAC probe and are displayed at respective BAC probe's center position (red circles). Shortest region of amplification overlap (SRO) was defined from involved BAC probes genomic start and stop position and is marked with a light gray background. Genomic position of eight BAC probes mapping to the SRO is displayed together with their center position (black circle). (**B) **Close-up of chr17:35000001-35200000 (hg18 build) for tumor TAX577717 analyzed by zoom-in oligonucleotide aCGH. (**C) **Close-up of chr17:35000001-35867695 (hg18 build) for tumor TAX577700 analyzed by zoom-in aCGH.

The 17q12-q21 amplification pattern was analyzed in 200 HER2+ tumors using BAC aCGH. The ability of the BAC aCGH platform to accurately estimate *HER2 *copy numbers was confirmed by parallel FISH analysis (Additional file 2) in 13 FFPE *HER2*-amplified tumors, showing a good correlation between the techniques (Figure S1A in Additional file 4). The smallest region of amplification overlap (SRO) for the *HER2 *amplicon was 248 kbp (chr17:34979166-35227087, hg17 build), involving 10 RefSeq genes (Figure 1A). The most frequently up-regulated genes (gene expression log_2_ratio ≥1) in this SRO were *HER2 *(92% of samples) followed by *GRB7 *(85%), *C17orf37 *(79%), *PERLD1 *(72%), *PPP1R1B *(63%) and *STARD3 *(62%), while gene expression data were unavailable for *NEUROD2*, *TCAP*, *PNMT *and *ZNFN1A3*. The *HER2 *SRO was further delineated by zoom-in aCGH to 85.92 kbp (chr17:35074472-35160391, hg18 build) including *TCAP*, *PNMT, PERLD1, HER2, C17orf37 *and *GRB7*. Thus, this analysis excluded *STARD3*, since three tumors showed an amplicon breakpoint within the gene, while one tumor had an amplicon starting immediately telomeric of *STARD3 *(Figures 1B and 1C).

*HER2/TOP2A *co-amplification was observed in 61 tumors (31%) with an SRO of approximately 1,050 kbp (chr17:34873000-35921000, hg17 build) using BAC aCGH, and narrowed to 783.64 kbp (chr17:35067680-35851322, hg18 build) using zoom-in aCGH (Figure 1C). *TOP2A *mRNA levels were significantly higher in HER2/TOP2A co-amplified cases as compared to non-amplified HER2+ tumors (*P *= 3 × 10^-5 ^and 7 × 10^-8^, respectively for TOP2A loss and TOP2A normal cases, t-test) in agreement with Arriola *et al. *[20], as well as compared to HER2- tumors, classified according to gene expression subtypes (for example, *P *= 0.0002 for HER2+/TOP2A+ vs. HER2- basal-like tumors, t-test) (Figure S1B in Additional file 4). Intriguingly, HER2+/TOP2A+ tumors showed significantly lower S-phase fractions and lower correlation to a gene expression grade signature [36] than HER2+/TOP2A- tumors (*P *= 0.004 and 0.001, respectively, t-test) (Figure S1B in Additional file 4). Additionally, *HER2/TOP2A *co-amplification was associated with ER+ status, as also observed by others [19,37], as well as patient age ≥50 years (*P *= 0.008 and 0.03, respectively, Fisher's exact test), but not to other clinical variables. Loss of *TOP2A *was found in 36 (18%) HER2+ tumors, while the remaining 51% had neither loss nor amplification of *TOP2A*. Moreover, none of 554 HER2- tumors had focal *TOP2A *amplification.

### Extent and patterns of significant CNAs on chromosome 17 in HER2+ breast cancer

**Figure 2:**
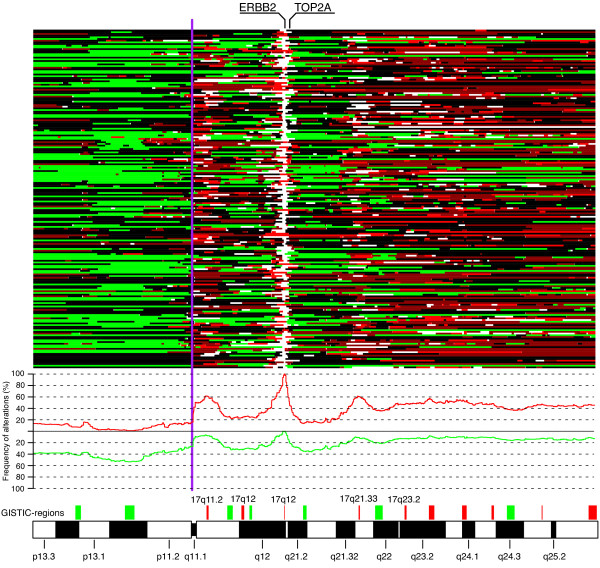
**Extent, frequency and patterns of CNAs on chromosome 17 in HER2+ BC**. Regions of loss are shown in green, normal in black, gain in dark red, amplification in red, and high-level amplification in white for each sample (row). Frequency of gain (red) and loss (green) across all 200 tumors are shown for chromosome 17. Read boxes, above the cytoband bar, indicate GISTIC regions of gain and green boxes GISTIC regions of loss. GISTIC regions with recurrent amplifications that are more frequent in HER2+ BC compared to HER2- BC are named. Vertical purple line corresponds to centromer limit.

Chromosome 17q has been reported to frequently harbor complex CNAs in HER2+ BC, often involving other high-level amplifications together with the 17q12 locus [18-20]. GISTIC analysis was used to identify and delineate 17 significant regions (10 gains, including the HER2 amplicon, and 7 losses) on chromosome 17 in the 200 HER2+ tumors (Figure 2, Additional file 5). Recurrent high-level amplifications were observed in 12 of 17 regions, of which amplifications on 17q11.2, 17q12 (centromeric of *HER2*), 17q12 (*HER2*), 17q21.33 and 17q23.2 (centromeric) were more prevalent in HER2+ compared to HER2- tumors. While 41% of all HER2+ tumors contained ≥1 other recurrent 17q amplicon besides *HER2*, no recurrent amplifications were identified on 17p. Several genes in the 17 GISTIC regions showed significant correlation between mRNA expression and copy number levels, including genes and miRNAs implicated in BC oncogenesis like *RPS6KB1 *[38], *PPM1D *[18] and *mir-21 *[39] on 17q23.2 (Additional file 5). *MYST2*, proposed as the candidate oncogene in the 17q21.33 region [40], was amplified in 20% of the tumors, however, located centromeric of the 17q21.33 GISTIC region. Besides the association of amplification on 17q11.2 with ER+ tumor status (*P *= 0.009, Fisher's exact test), none of the other 17q amplicons were significantly correlated with ER status, LN status, tumor size, histological grade or patient age, possibly due to the small sample numbers for individual amplicons.

Amplification of centromeric regions on chromosome 17 (CEP17 amplification) was observed in 22 (11%) tumors using the closest pericentromeric BAC probes. CEP17 amplification based on copy number status for BAC probes on either 17p11.1 or 17q11.1 was found in 37 (19%) HER2+ tumors. By comparison, only four (1%) HER2- tumors in the Jönsson *et al. *data set showed CEP17 amplification based on either pericentromeric BAC probes, or average copy number of BAC probes on either 17p11.1 or 17q11.1. Zoom-in aCGH analysis further delineated the pattern of amplification in the centromeric region, identifying amplification of a region including the *WSB1 *gene (chr17:22558232-22751802, hg18) as the most frequent in tumors with CEP17 amplification.

### Recurrent amplifications and firestorm-like amplification patterns in HER2+ breast cancer

**Figure 3:**
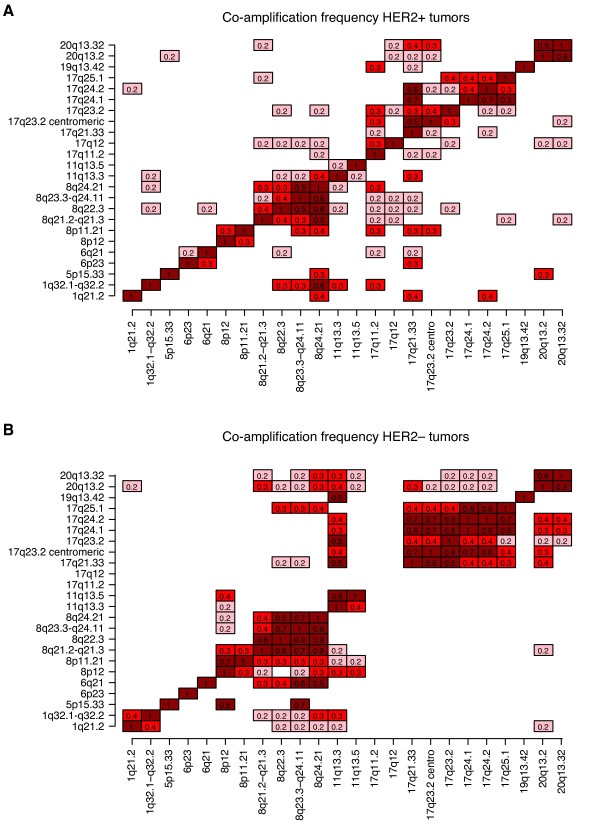
**Pattern of co-occurrence of recurrent amplifications in HER2+ and HER2- BC**. (**A**) Fraction of co-amplification of recurrent amplifications in HER2+ BC excluding the 17q12 HER2 locus. For each amplification (vertical axis), the fraction of samples with a co-amplification (horizontal axis) is indicated in each box. Only co-amplifications occurring in ≥2 tumors with fractions ≥0.2 are displayed. For example, 20% of tumors with 17q24.2 amplification also have 1q21.2 amplification, while 40% of tumors with 1q21.2 amplifications also show amplification at 17q24.2 indicating that the number of 1q21.2 amplified tumors is lower than the number of tumors with 17q24.2 amplification. (**B**) The fraction of co-amplification of recurrent amplifications in A in HER2- breast tumors. Only co-amplifications occurring in ≥3 tumors with fractions ≥0.2 are displayed. Fractions are calculated similarly as in A.

Excluding chromosome 17, recurrent high-level amplifications were observed with varying frequencies on chromosomes 1, 5, 6, 8, 11, 19 and 20 in 90 (45%) of HER2+ tumors (Additional file 6). Interestingly, there was no significant difference in their overall prevalence in HER2+ and HER2- tumors (*P *> 0.05, Bonferroni adjusted Fisher's exact test). Identified amplifications included several known BC amplicons and oncogenes, for example, 8p12 (*FGFR1*, *LSM1*, *RAB11F1P1*, *PPAPDC1B*), 8q24.21 (*MYC*), 11q13.3 (*CCND1*) and 20q13.2 (*ZNF217*). Co-occurrence of amplicons was also common as 30%, 9% and 8% of the 90 tumors had two, three or more than three recurrent amplifications, respectively. A number of chromosome regions were frequently co-amplified: amplifications on 8q (one with another), most regions on 17q (with each other), 1q32.1-q32.2 with 8q24.21, and 20q13.2 with 20q13.32 (Figure 3A). Interestingly, while high-level amplifications of 8p12, 11q13.3 and 20q13.2 were mutually exclusive in the HER2+ tumors, these co-amplifications were not uncommon in HER2- tumors (Figure 3). Furthermore, co-occurrence of high-level amplifications at 8p12 with 8q24.21, and 20q13.2 with 8q24.21 were also rare in HER2+ tumors compared to HER2- tumors (Figure 3). High-level amplifications of other putative oncogenes, for example, *PIK3CA*, *ESR1*, *EGFR, KIT, MDM2 *and *MYB*, were rare in HER2+ tumors (≤1%). Amplifications on 11q13.3, 11q13.5 and 19q13.42 were associated with ER+ tumor status (*P *= 0.002, 0.06, 0.03 respectively, Fisher's exact test), corroborating previously reported association of 11q13.3 to HER2+/ER+ disease [19]. No recurrent amplification was associated with LN status, tumor size or patient age.

A firestorm-like amplification pattern (firestorms) has been defined as multiple closely spaced high-level amplifications limited to single chromosome arms [30]. In total, 115 firestorms were observed in 88 (44%) HER2+ tumors. Firestorms observed on p-arms (n = 14) were predominantly located on 1p (36%), 6p (21%) and 12p (14%), while firestorms observed on q-arms (n = 101) were predominantly located on 17q (57%), 8q (16%), 20q (5%), 6q (4%), 12q (3%), and 1q (3%). By comparison, a total of 39 firestorms were observed in 30 (10%) HER2- tumors in the Jönsson *et al. *data set analyzed similarly on the same BAC aCGH platform. Firestorms in these HER2- tumors were predominantly located on 1q, 6q, 8q, 11q, 12p, 12q, and 17q (>1 firestorm). Amplification peaks in observed firestorms were rarely recurrent across HER2+ tumors, except for a few peaks on 17q that were observed in multiple tumors. Prevalence of firestorms was correlated with LN+ status and DNA aneuploidy in HER2+ tumors (*P *= 0.02 and 0.009, respectively, Fisher's exact test), but not to ER status or tumor size.

### Comparison of DNA copy number alterations in HER2+ and HER2- breast cancer

**Figure 4:**
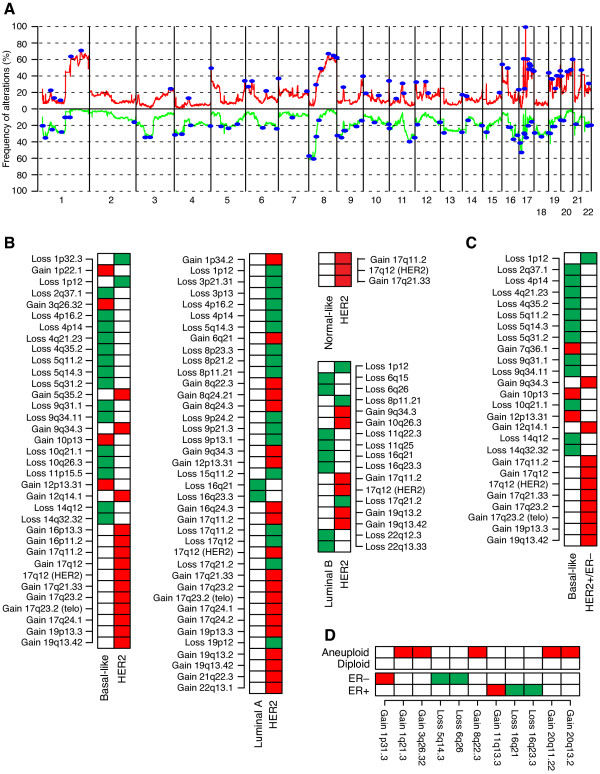
**Significant CNAs in HER2+ BC in relation to molecular subtypes**. (**A**) Frequency of gain (red) and loss (green) in 200 HER2+ tumors. Blue regions indicate significant CNAs identified by GISTIC analysis. (**B**) GISTIC regions differing HER2+ tumors from HER2- tumors classified according to the molecular subtypes as basal-like, luminal A, luminal B and normal-like (*P *< 0.05, Bonferroni adjusted Fisher's exact test). Each box represents a GISTIC region, red indicates more frequent gain, and green indicates more frequent loss. (**C**) GISTIC regions differing HER2+/ER- tumors from HER2- tumors classified as basal-like. Regions identified by Bonferroni adjusted Fisher's exact test (*P *< 0.05). Each box represents a GISTIC region, red indicates more frequent gain, and green indicates more frequent loss. (**D**) GISTIC regions associated with ER status and DNA ploidy in HER2+ BC. Regions identified by Student's t-test with FDR-adjusted *P *< 0.05. Each box represents a GISTIC region, red indicates more frequent gain, and green indicates more frequent loss.

HER2+ tumors revealed considerable genomic heterogeneity with the most frequent CNAs (>30% of tumors) being gain on 1q, 5p, 6p, 8q, 9q, 11q, 12p, 12q, 16p, 17q, 19p, 19q, 20p, 20q and 21q, and loss on 1p, 3p, 8p, 9p, 11q, 16q, 17p, 17q and 18q (Figure 4A). GISTIC analysis identified 117 regions (58 gains, 59 losses), located across all autosomes including numerous candidate genes and miRNAs (Figure 4A, Additional file 5).

Several GISTIC regions showed significant differences in frequency when HER2+ tumors were compared to HER2- tumors classified into gene expression subtypes (Table 1, Figure 4B, Additional file 7). Basal-like tumors were characterized by more frequent losses on 2q37.1, 4p, 4q, 5q, 9q, 10q, 11p15.5 and 14q, and gain of 1p22.1, 3q26.32, 10p13 and 12p13.31 compared to HER2+ tumors. Luminal A tumors were characterized by more frequent losses on 16q, while other GISTIC regions were less frequently altered compared to HER2+ tumors. Luminal B tumors were characterized by more frequent losses on 6q, 11q, 16q and 22q compared to HER2+ tumors, while normal-like tumors were characterized by an overall lower CNA frequency.

The average fraction of the genome altered (FGA), representing the percentage of BAC clones subjected to gain or loss for each sample, for HER2+ tumors was 0.34, equally divided between gains (0.18) and losses (0.16). HER2+/ER- tumors showed significantly lower FGA than HER2-/ER- tumors (*P *= 9 × 10^-11^, t-test), while HER2+/ER+ tumors were not different from HER2-/ER+ tumors (*P *= 0.11). A similar comparison of HER2+ tumors to HER2- tumors of various gene expression subtypes showed significantly higher FGA in basal-like (*P *< 2 × 10^-16^) and luminal B (*P *= 0.0001) tumors, but lower FGA in luminal A (*P *= 0.0003) and normal-like tumors (*P *= 0.04) compared to HER2+ tumors.

### Differences between HER2+ and basal-like breast cancer

Since most ER- tumors are found in the ERBB2 and basal-like gene expression subtypes [15,41], we performed separate comparisons of HER2+/ER- tumors vs. HER2- basal-like tumors, and ERBB2 classified tumors vs. HER2- tumors classified according to gene expression subtypes. Comparison of HER2+/ER- tumors vs. basal-like tumors resulted in similar findings as for all HER2+ tumors vs. basal-like tumors (Figure 4C). Moreover, comparison of ERBB2 classified HER2+ tumors (n = 51) to basal-like tumors identified similar regions as in the comparison of HER2+/ER- tumors (Additional file 8). We were not able to confirm findings that loss of 15q14-q21 and 9p21.3 separate basal-like tumors from HER2+ tumors [18]. In addition, HER2+ tumors displayed differences in mRNA expression of two different ER gene expression modules [42,43] compared to HER2- tumors classified according to the gene expression subtypes in the Jönsson *et al. *data set (Additional file 9). Notably, basal-like tumors displayed considerably lower expression of the ER gene expression modules compared to HER2+/ER- tumors, while the difference between HER2+/ER- and HER2+/ER+ tumors was less pronounced.

### DNA copy number alterations in subgroups of HER2+ breast cancer

Highly similar CNA frequencies were observed when HER2+ tumors were stratified by ER status, with apparent differences being limited to more frequent loss of 1p, 11q and 16q and gain of 11q13 in HER2+/ER+ tumors, and loss of 5q in HER2+/ER- tumors (Additional file 10). Supervised analysis of subgroups of HER2+ tumors defined by clinical or tumor biomarkers identified 11 GISTIC regions significantly associated with ER status or DNA ploidy (Figure 4D). Several GISTIC regions (for example, +1p31.3, - 5q14.3, +11q13.3, - 16q23.3) separated ER+ from ER- tumors in HER2+ BC, but their effect was evident also in HER2- tumors (*P *= 0.06, 5 × 10^-23^, 0.0002, 0.0003 respectively in HER2- tumors, Bonferroni adjusted t-test). No GISTIC regions separated HER2+ tumors stratified by patient age, LN status, tumor size, or histological grade 1 or 2 vs. 3. Differences in FGA were observed for HER2+ tumors stratified by LN status (*P *= 0.02, t-test) and DNA ploidy (*P *= 1 × 10^-8^), but not by ER status, tumor size, histological grade or patient age.

Of the 200 HER2+ tumors analyzed by BAC aCGH, 87 had concurrent gene expression data and were classified according to the gene expression subtypes [15] (Table 1). Notably, 24% of tumors classified to the ERBB2 subtype were ER-positive. The individually small subtype groups prevented individual pair-wise comparisons with the ERBB2 subtype. However, no significant GISTIC regions, and no significant difference in FGA were found in a pair-wise comparison of the tumors in the ERBB2 subtype (n = 51) vs. tumors in remaining expression subtypes combined (n = 30). Strikingly, 83% of HER2+/ER- cases with available gene expression data were classified into either the basal-like or ERBB2 subtype, while only 30% of HER2+/ER+ tumors were classified to any of the two luminal subtypes.

### Associations of histopathological and genomic characteristics with overall survival

LN status, DNA ploidy and tumor size were independent significant variables for OS in 176 patients with primary HER2+ BC, while ER status, patient age and histological grade were not associated with OS (Table 2). HER2+ cases classified to the ERBB2 gene expression subtype have been reported to show a tendency for poorer relapse-free survival compared to HER2+ cases classified to the other subtypes [44]. However, we were not able to verify such a prognostic association using OS as an endpoint in the current study, for either all five subtypes separately, or the ERBB2 subtype vs. remaining four subtypes combined (data not shown).

**Table 2:** Log-rank, univariate and multivariate associations with OS for clinical variables and genomic characteristics for 176 primary HER2+ tumors

Investigated covariate^a^	Number tumors	Log-rank *P*	Univariate *P*	Multivariate *P* ^b^
ER+ vs. ER-	64/112	0.96	0.96	0.79
**LN+ **vs. LN-	110/65	4 × 10^-6 ^***	9 × 10^-6 ^***	0.0002***
Size **>20 mm **vs. ≤ 20 mm	122/49	0.002**	0.002**	0.02*
Histological grade 3 vs. 1 and 2	37/26	0.91	0.91	0.83
Age <50 vs. ≥50 years	73/103	0.22	0.23	0.77
DNA **Aneuploid **vs. Diploid	106/37	0.001**	0.001**	0.005**
DNA **Aneuploid **vs. Diploid ER+	37/11	0.008**	0.02*	0.07
DNA **Aneuploid **vs. Diploid ER-	69/26	0.05*	0.05*	0.06
Recurrent 17q amplification, yes vs. no	70/106	0.17	0.17	0.13
Recurrent amplification excluding 17q, yes vs. no	76/100	0.75	0.75	0.7
Firestorm pattern, yes vs. no	78/98	0.27	0.28	0.58
High FGA vs. low FGA^c^	42/46	0.08	0.09	0.25
High FGA vs. low FGA ER+ tumors^c^	18/17	0.09	0.09	0.26
High FGA vs. low FGA ER- tumors^c^	24/29	0.37	0.37	0.55

^a ^Variables with worst outcome highlighted in bold for covariates significantly associated with OS.^b ^Multivariate analysis included LN status and stratified tumor size besides the tested covariate.^c ^High and Low FGA defined as >75^th ^percentile and <25^th ^percentile of all FGA values respectively.

*: Significant at *P *< 0.05

**: Significant at *P *< 0.01

***: Significant at *P *< 0.001

**Figure 5:**
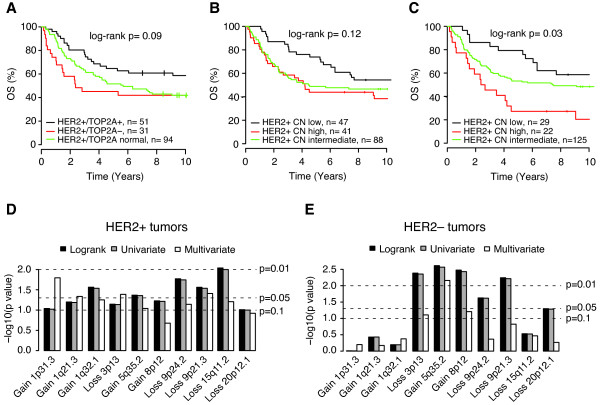
**Association of OS with TOP2A-status, *HER2 *copy number levels, and GISTIC regions in HER2+ and HER2- tumors**. (**A**) OS in primary HER2+ tumors stratified by TOP2A-status. (**B**) OS in primary HER2+ tumors stratified by the 25^th ^(HER2+ CN low) and 75^th ^percentile (HER2+ CN high) of the mean *HER2 *segmented log_2_ratio. (**C**) OS in primary HER2+ tumors stratified by the 15^th ^(HER2+ CN low) and 85^th ^percentile (HER2+ CN high) of the mean *HER2 *log_2_ratio. (**D**) GISTIC regions showing association with OS (log-rank *P *< 0.1) in the 176 primary HER2+ tumors. The vertical axis represents -log_10_(p) for log-rank, univariate and multivariate analysis. Tumor size and LN status were included as covariates in multivariate analyses beside GISTIC regions. Dashed lines indicate *P *= 0.1, 0.05 and 0.01. GISTIC regions are ordered according to their genomic position. (**E**) Association to OS for GISTIC regions in D in HER2- tumors. The vertical axis represents -log_10_(p) for log-rank, univariate and multivariate analysis. Tumor size and LN status were included as covariates in multivariate analyses besides GISTIC regions. Dashed lines indicate *P *= 0.1, 0.05 and 0.01. GISTIC regions are ordered according to their genomic position.

There was a trend towards a different outcome for patients stratified according to TOP2A status (Figure 5A), and stratification by *HER2 *copy number estimates showed that patients with the highest *HER2 *copy number estimates had significantly worse OS compared to tumors with the lowest estimates (Figures 5B and 5C). However, the difference in OS for the latter case is at least partly explained by that cases in the group with the highest *HER2 *copy numbers were more frequently DNA aneuploid (*P *= 0.1, Fisher's exact test) and displayed higher FGA values (*P *= 0.04, t-test) compared to cases in the group with the lowest copy numbers. No association with outcome was seen for the presence of recurrent amplifications or firestorms in patients with primary HER2+ BC. This lack of association remained significant also when stratifying patients with DNA aneuploid or diploid HER2+ tumors for presence of recurrent amplifications or firestorms respectively (data not shown). In contrast, patients with HER2- tumors displaying a firestorm-like amplification pattern in the Jönsson *et al. *data showed significantly worse OS (log-rank *P *= 0.0008) supported by multivariate analysis (n = 250, *P *= 0.002, HR = 2.3, 95% confidence interval (CI) = 1.4 to 3.8). A tendency for worse OS was observed for HER2+ cases with high vs. low FGA (log-rank *P *= 0.08) especially for HER2+/ER+ tumors (Table 2). The association was however weakened considerably when FGA was stratified for DNA aneuploidy (log-rank *P *= 0.52 for all tumors and *P *= 0.65 for HER2+/ER+ tumors), while DNA aneuploidy still added prognostic information when stratified for FGA (log-rank *P *= 0.02 for all tumors, *P *= 0.07 for HER2+/ER+ tumors). Low sample numbers hampered the investigation of individual recurrent amplifications, but 10 GISTIC regions showed moderate association to OS (log-rank *P *< 0.1) when comparing gain or loss vs. normal copy number in HER2+ cases (Figure 5D). Three of these regions (-3p.13, +5q35.2 and +8p12) were also associated with OS in the combined HER2- reference data set (log-rank *P *= 0.004, 0.002 and 0.003, respectively) adding either independent or near independent prognostic information in multivariate analysis (*P *= 0.08, 0.007 and 0.06, respectively) (Figure 5E). Of these regions, 8p12 has previously been identified as an indicator of poor breast cancer prognosis [31]. The association with outcome for the 5q35.2 GISTIC region was stronger for HER2-/ER- tumors compared to HER2-/ER+ tumors (log-rank *P *= 0.006 and 0.07 respectively).

### Correlation of gene expression data with genomic aberrations

Gene expression data were compared with genomic aberrations in 87 HER2+ tumors in order to identify genes affected by gene dosage. First, genes in the 117 GISTIC regions (n = 1,750) were matched to available gene expression data (n = 1,078) and correlated across tumor samples as previously described [25]. This approach identified 460 significantly correlated genes, 284 and 176 in GISTIC regions of gain and loss, respectively (Additional file 5). Second, analysis was re-performed without the restriction to genes in GISTIC regions. Of 10,162 matched genes, 5,853 genes (58%) showed a standard deviation >0.5 in mRNA expression, and 2,242 (38%) of these genes showed significant correlation between expression and copy numbers (*P *< 0.05 adjusted for multiple testing) (Additional file 11). In summary, correlation of mRNA and copy number identified numerous breast cancer tumor suppressor genes and oncogenes, such as *STAT5A*, *SNIP*, *ZNF217*, *TOP2A*, *BCL2, PAK1*, *PER3*, *CCND1*, *NME1*, *PPAPDC1B*, *LSM1*, *IL17RB *and *FGFR1*, to be significantly correlated in HER2+ tumors. In addition, genes previously reported to be significantly correlated in HER2+/TOP2A co-amplified cases (for example, *CASC3*, *CDC6*, *RARA *and *SMARCE1*) [20] were also identified in this study (Additional file 11).

## Discussion

We have characterized a large set of HER2+ BC in comparison to HER2- BC using a combination of molecular techniques to delineate the *HER2*-amplicon in high-detail, and to pinpoint, on a genome-wide scale, critical regions of focal amplifications, gains and losses that may be important for tumor development and reflect the heterogeneity of HER2+ BC.

### The HER2-amplicon

By using a custom-designed zoom-in aCGH platform we delineated the shortest region of overlapping amplification for the *HER2*-amplicon to an 85.92 kbp region including six genes. The identified region is considerably smaller than previously reported [19,20,45], mainly due to the larger number of tumors and the extreme probe density provided by the zoom-in aCGH platform. While the role of some amplicon genes may be less relevant for breast cancer development, the function of *GRB7 *is intriguing. *GRB7 *showed strong correlation between mRNA transcript levels and copy number status in this study, and increased mRNA expression has been shown to correlate with protein overexpression in breast cancer cell lines [46,47]. As an SH2-containing adapter protein GRB7 can interact with phosphorylated HER2 and mediate aspects of cell migration through binding with focal adhesion kinase [48,49]. Furthermore, *GRB7 *has been pinpointed as one of the top-ranked genes in a HER2-derived prognostic gene signature [17], arguing that it is not merely a silent passenger of the amplicon. However, we found no case of focal *GRB7 *amplification in HER2- cases suggesting that its activation is linked to the selected advantage conferred by *HER2 *activation.

### CEP17 amplification in HER2+ breast cancer

Accurate evaluation of *HER2 *status is important for identification of patients that would benefit from HER2 targeted therapy. FISH analysis for determination of the ratio of *HER2 *copy number to chromosomes 17 copy number, represented by a centromeric chromosome 17 FISH probe (*HER2*/CEP17 ratio), has been suggested as the current golden standard [50-52]. However, concerns have been raised, based on aCGH and MLPA studies, whether CEP17 copy number status accurately reflects true chromosome 17 copy number and polysomy (≥3 copies of the entire chromosome 17) [53-55]. We found that polysomic chromosome 17, using the definition suggested by Marchio *et al. *[53], was a rare event in HER2+ breast tumors (1.5% of tumors). While CEP17 amplification was a rare event in HER2- tumors, it was observed in a much higher frequency in HER2+ tumors, consistent with recent reports [14]. Thus, our data support the notion that abnormal CEP17 copy numbers more likely stem from CNAs on chromosome 17q, and that CEP17 correction may for certain cases be misleading for interpretation of HER2 status [53,54].

### TOP2A aberrations in HER2+ breast cancer

The variable structure of the HER2 amplicon frequently involves additional genes telomeric of *HER2 *[20,56], for instance *TOP2A *encoding a protein target of anthracyclines [57]. It has become increasingly evident that *TOP2A *alterations rarely occur in HER2- breast tumors (reviewed in [9]) in line with our finding that no HER2- tumor showed focal amplification of *TOP2A*. In this study, *TOP2A *amplification or deletion was observed in 31% and 18% of HER2+ tumors, respectively, concordant with previous reports (reviewed in [9]). Consistent with previous reports [56], we found in tumors analyzed by high-resolution zoom-in aCGH that co-amplification of *HER2 *and *TOP2A *was not separated by chromosomal regions with normal or deleted copy numbers, and that discordance between *HER2 *and *TOP2A *copy numbers existed in co-amplified cases. The strong correlation between *TOP2A *copy number and expression level (Figure S1B in Additional file 4, Additional file 11) and the abrupt breaks in the *HER2*/*TOP2A *amplicon telomeric of *TOP2A *in several tumors (for example, Figure 1C), suggest a selective retention of *TOP2A *activation in the development of some tumors through, for instance, breakage-fusion-bridge cycles [56]. *TOP2A *alterations clearly have a potential role in tumor progression and treatment response, and have been linked to better disease-free survival for patients with HER2+ disease treated with anthracyclines [11,37]. However, TOP2A protein expression has been reported to correlate more with cellular proliferation than gene amplification [58], in line with our findings of elevated *TOP2A *mRNA levels in highly proliferative HER2- basal-like and luminal B tumors for which no focal *TOP2A *amplification was observed (Figure S1B in Additional file 4). Moreover, it has recently been suggested that alterations in the centromeric region of chromosome 17 is a more powerful predictor of response to anthracycline-based treatment than alterations in either *HER2 *or *TOP2A *[14]. Taken together, the finding of a trend of better OS for HER2+/TOP2A+ tumors in the present study is difficult to interpret, as we had no specific treatment information available for patients in this study. Clearly, the complex relationship of individual genes in the 17q12-q21 region, as well as other genomic alterations on chromosome 17q, to breast cancer development and treatment efficacy warrants further investigation.

### Recurrent amplifications and firestorm patterns in HER2+ breast cancer

Recurrent high-level amplifications and firestorms were frequent in HER2+ breast tumors as also observed by others [18-20]. In line with previous reports, HER2+ tumors were firmly associated with an amplifier/firestorm-like genomic pattern, as firestorms or recurrent amplifications, excluding the *HER2*-amplicon, were observed in 70% of all HER2+ tumors [18,30,31]. However, only a few recurrent amplifications on chromosome 17q were more common in HER2+ than in HER2- tumors. In contrast to recent reports [30,31], presence of firestorms or recurrent high-level amplifications were not associated with clinical outcome for patients with HER2+ breast tumors. Instead, DNA aneuploidy was a stronger indicator of poor prognosis, especially for HER2+/ER-positive tumors, in line with previous reports for breast cancer irrespective of HER2 status [59,60]. Although firestorms were significantly more frequent in DNA aneuploid HER2+ tumors, 25% of DNA diploid HER2+ cases displayed firestorms suggesting that occurrence of gross chromosomal alterations and amplifier patterns may be unrelated mechanisms of genomic instability.

The majority of identified recurrent amplifications in this study have previously been reported in both HER2+ and HER2- breast tumors, although with different frequencies and co-amplification patterns [18,31,32,38]. A few discrepancies exist in comparison to recent aCGH studies on HER2+ tumors, for example, we did not observe recurrent (>2%) high-level amplifications on chromosome 7p, 14q and 18q [18,20]. Interestingly, while combinations of high-level amplifications of 8p12, 11q13.3 and 20q13.2 were not uncommon in HER2- tumors, they were mutually exclusive in HER2+ tumors. When the threshold for high-level amplification was lowered to that of amplification, co-occurrence of these regions was still rare in HER2+ tumors compared to HER2- tumors (data not shown). Individual amplification of 8p12, 11q13.3, or 20q13.2 was associated with worse OS in HER2- tumors (log-rank *P *≤ 0.02), but not in HER2+ tumors. Furthermore, co-amplification of 11q13.3 with 8p12 and 11q13.3 with 20q13.2 remained significantly associated with outcome in HER2- tumors despite fewer cases, while co-amplification of 8p12 with 20q13.2 showed only a trend (log-rank *P *= 0.10). Presumably, these amplifications activate cellular pathways that drive tumor progression synergistically in HER2- tumors, whereas having more than one of these amplifications provides no advantage in HER2+ tumors.

### Significant CNAs in HER2+ breast cancer

The molecular subtypes of breast cancer [15] have been associated with distinct CNAs and genomic patterns [18,31,38,61] that may contribute to their transcriptional profiles and biological phenotypes [62]. The overall pattern of CNAs in HER2+ breast tumors observed in this study corroborates earlier findings [18,19]. However, a few discordances exist, mainly corresponding to more frequent gain of 16p and less frequent aberrations on chromosome 7 in the current study. Comparison of CNAs in HER2+ tumors to HER2- tumors revealed differences in FGA and frequency of several GISTIC regions (Figure 4, Additional files 5, 7, 8). On the other hand, some of the most recurrent CNAs in HER2+ tumors irrespective of ER status, including +1q, +8q, -8p, and -17p, were also commonly observed in HER2- tumors indicating their importance in breast cancer. Several of the regions discriminating HER2+ from HER2- tumors have previously been reported as specific for basal-like, luminal A and B tumors respectively [31-33,40,61,63,64]. However, certain discriminatory regions are explained by the ER status of the 200 HER2+ tumors and the gene expression subtypes. For instance, HER2- luminal A and B tumors displayed more frequent loss of 16q than HER2+ tumors as a whole (Figure 4B). However, when HER2+/ER+ tumors were compared to luminal A and B tumors, loss of 16q was no longer significant, reflecting the strong association of specific CNAs with ER status (data not shown). Furthermore, our data, supported by subtype classifications of independent breast cancer data sets, indicate that the ERBB2 subgroup, although dominated by HER2+/ER- tumors, contains a sizeable fraction of HER2+/ER+ tumors.

The finding of GISTIC regions stratifying both HER2+ and HER2- tumors based on ER status is in contrast to a recent smaller aCGH study on HER2+ breast tumors [19]. Interestingly, although HER2+/ER- tumors harbor a pattern of CNAs similar to HER2-/ER- and basal-like tumors, the frequencies of these aberrations are significantly lower in HER2+/ER- tumors as well as ERBB2 subtype classified HER2+ tumors, in agreement with Marchio et al. [19] (Figure 4C, Additional files 5 and 8). HER2+/ER- tumors have been linked by gene expression analysis to an apocrine/steroid response-positive subgroup of ER-negative BC characterized by overexpression of genes related to steroid estrogen response [65,66]. Moreover, there is increasing support for crosstalk between the HER2 and ER-signaling pathways (reviewed by [67,68]). Consistent with these observations and recent reports [42,69], HER2+ tumors in the current study showed intermediate expression of two ER gene expression modules, which were significantly less expressed in HER2- basal-like tumors. Further substantiating the difference between HER2+/ER- and HER2- basal-like tumors we did not find elevated frequencies of CNAs characteristic of HER2- basal-like BC in HER2+ tumors with high correlation to the basal-like gene expression centroid (data not shown).

In summary, HER2+ tumors display a wide range of frequently complex CNAs including firestorms and recurrent amplifications. However, with the exception of a limited number of CNAs primarily located on chromosome 17q, the vast majority of CNAs does not appear specifically associated with HER2+ tumors per se, as revealed when compared to other breast cancer subgroups. These findings underline the genomically complex and heterogeneous nature of HER2+ breast cancer in relation to other subgroups of breast cancer.

## Conclusions

We have conducted a comprehensive survey of copy number alterations in HER2+ breast tumors using a combination of aCGH and gene expression analysis, pinpointing significant genomic alterations including both known and potentially novel therapeutic targets. Our analysis sheds further light on the genetically complex and heterogeneous nature of HER2+ tumors in relation to other breast cancer subgroups.

## Abbreviations

aCGH: array-based CGH; BAC: Bacterial artificial chromosome; BC: breast cancer; CBS: circular binary segmentation; CEP17: chromosome 17 centromere; CNA: copy number alteration; ER: estrogen receptor; FFPE: formalin fixed paraffin embedded; FGA: fraction of the genome altered; FISH: fluorescence in situ hybridization; GISTIC: Genomic Identification of Significant Targets in Cancer; IHC: immunohistochemistry; LN: lymph node; OS: overall survival; SRO: shortest region of overlap.

## Competing interests

NL has received honoraria from Roche and AstraZeneca. The other authors declare that they have no competing interests.

## Authors' contributions

ÅB and JS conceived of the study. JS, GJ, AA and HG performed array CGH experiments. DG performed FISH and IHC experiments. JS performed data analysis with support by MR and GJ. JS wrote the manuscript with the assistance of GJ, MR, JVC and ÅB. BA, NL, OJ, PM, RB and DG contributed samples and clinical information. All authors approved the final manuscript.

## Supplementary Material

Additional file 1 **Patient and tumor characteristics for *HER2*-amplified tumors**. An Excel table containing clinical and experimental data on the 200 *HER2*-amplified tumors. Click here for file

Additional file 2 **Supplementary methods**. A Word document containing supplementary information about used methods and data processing. Click here for file

Additional file 3 **Patient and tumor characteristics for *HER2*-negative tumors**. A Word document containing a table of clinical data for HER2- tumors in the reference breast cancer data set. Click here for file

Additional file 4 ***HER2 *copy number evaluation and *TOP2A *mRNA expression levels**. A pdf file containing figures of the result of the comparison of *HER2 *copy number estimates between aCGH and FISH for 13 FFPE samples (S1A) and mRNA expression levels and S-phase fractions for HER2+ and HER2- tumors in the Jönsson et al. data set (S1B). Click here for file

Additional file 5 **Significant GISTIC regions in *HER2*-amplified breast cancer**. An Excel table presenting the 117 GISTIC regions, including genes significantly correlated between mRNA expression levels and copy numbers, and frequency of high-level amplifications on chromosome 17q. Additionally, the frequency of GISTIC regions in HER2+ tumors overall and stratified by ER status is presented and compared to corresponding frequencies in HER2- tumors stratified by ER status. Click here for file

Additional file 6 **Recurrent high-level amplifications in *HER2*-amplified breast cancer**. A Word file describing recurrent high-level amplifications, excluding chromosome 17, in HER2+ breast tumors including genes in amplicons significantly correlated between mRNA expression levels and copy numbers. Click here for file

Additional file 7 **Frequency of GISTIC regions in *HER2*-amplified and *HER2*-negative breast cancer**. An Excel table containing the results from the comparison of frequencies of the identified GISTIC regions in *HER2*-amplified breast cancer compared to HER2- tumors classified according to gene expression subtypes. Click here for file

Additional file 8 **Expression of ER gene expression modules in *HER2*-amplified and *HER2*-negative breast cancer**. A pdf file containing two subpanels illustrating: (1) differences in expression of two ER gene expression modules in the Jönsson *et al. *data set for HER2+ tumors stratified according to ER status, and (2) HER2- tumors classified according to gene expression subtypes. Click here for file

Additional file 9 **Frequency of GISTIC regions in *HER2*-amplified and *HER2*-negative breast cancer according to gene expression subtypes**. A pdf file containing two panels illustrating: (1) GISTIC regions significantly different between HER2+ tumors classified to the ERBB2 gene expression subtype, compared to HER2- tumors classified as basal-like, luminal A, luminal B and normal-like subtype, and (2) CNA frequency in HER2- basal-like classified tumors. Click here for file

Additional file 10 **Copy number alterations in *HER2*-amplified breast cancer stratified by ER status**. A pdf file containing two subpanels illustrating CNA frequencies in HER2+/ER- tumors and HER2+/ER+ tumors, respectively. Click here for file

Additional file 11 **Global correlation of mRNA expression and gene dosage in *HER2*-amplified breast cancer**. An Excel table listing genes that are found significantly correlated between gene expression data and aCGH in HER2+ tumors using a global matching. Click here for file
