# Supplementary material for: High-resolution genomic and expression analyses of copy number alterations in HER2-amplified breast cancer
Source: Breast Cancer Res. 2010 May 6;12(3):R25. doi: 10.1186/bcr2568 (PMC2917012; doi:10.1186/bcr2568)
Supplement: Additional file 2 — Supplementary methods. A Word document containing supplementary information about used methods and data processing. [file bcr2568-S2.DOC]

## Supplementary Material and Methods

# Isolation and quality assessment of DNA

#### DNA from fresh frozen tumor tissue was extracted using Proteinase K treatment followed by repeated phenol-chloroform extraction. DNA from FFPE tissue was extracted according to a reported protocol from the Array Core Facility at UCSF Helen Diller Family Comprehensive Cancer Center. FFPE DNA was quality controlled and in certain cases amplified using the Enzo BioScore kit according to manufacturer’s instructions (Enzo LifeSciences Inc., Farmingdale, NY).

# FISH – aCGH comparison on formalin-fixed paraffin embedded tissue

*HER* gene log_2_ratios for 13 HER2+ breast tumors were calculated from FISH ratios and aCGH. aCGH was performed on 32K BAC arrays with additional BAC clones covering the 17q12 amplicon (Gene Expression Omnibus platform GPL7247). For aCGH, estimates were calculated as the average log_2_ratio of seven BAC clones covering *HER2* (RP11-94L15, RP11-62N23, RP11-563O4, RP11-689B15, RP11-610O22, RP11-909L6 and RP11-1065L22) after lowess normalization. FISH was performed using the Dako HER2 FISH pharmDx^TM^ Kit Code no. K5331 (Dako, Glostrtup, Denmark) according to manufacturers’ instructions. FISH ratios were calculated as *HER2* red signals divided by chromosome 17 green signals in 20 nuclei and subsequently log_2_-transformed. Additionally, tumors were scored by immunohistochemistry (IHC). IHC was performed using HercepTest^TM^ for Techmate^TM^ Instruments Code no. K5206 (Dako, Glostrup, Denmark) according to manufacturers’ instructions. Scoring was performed as described in the HercepTest^TM^ Interpretation Manual (Dako).

#### Zoom-in aCGH analysis

Zoom-in aCGH microarrays aimed at chromosome 17 were designed using the online Agilent eArray ver 5.3 software. Probes were mapped to the hg18 build [24]. For the region chr17:34946000-36150819 all available probes in the eArray database (n=13346) were selected. For the region chr17:33730320-34946000 all exonic probes (n=4260) were selected. 1787 probes were selected for the centromeric region chr17:21200000-25400000. For the remaining part of chromosome 17q, probes were selected to obtain an average resolution of 2800bp. A probe set of 4368 60-mer oligonucleotides spaced at 500kbp throughout chromosomes 1-12, 16 and 17p was used for data centering. Array format was set to 4x44K.

*Identification of CEP17 BAC probes*

RP11-423O14, RP11-399C2, and RP11-29L17 were identified as the three BAC probes closest to the chromosome 17 centromer on the p-arm. RP11-260A9, RP11-102E1, and RP11-1432H15 were identified as the three BAC probes closest to the chromosome 17 centromer on the q-arm.

*Identification of significant copy number alterations using GISTIC*

The GISTIC [29] implementation used to detect significant copy number alterations in the 200 *HER2*-amplified tumors used a fixed log_2_ratio threshold of ± 0.1 for calculation of the G-score. Consequently, prior to GISTIC analysis CBS segmented log_2_ratios for each sample was rescaled so that an individual sample’s sample adaptive threshold (SAT) (Additional File 1) was set to ± 0.1. For each tumor, gain then corresponds to a GISTIC log_2_ratio>0.1 and loss to < -0.1 in rescaled log_2_ratio. CNV masking was performed by matching BAC probes to CNV data for the NCBI build 35 using the Toronto Database of Genomic Variants. GISTIC Regions were defined as GISTIC wide-peak limits and mapped to the hg17 build [24]. In *HER2*-amplified tumors, Student’s t-tests on average log_2_ratios for GISTIC regions were used to identify regions associated with different clinical variables. A false discovery rate-adjusted (FDR) p-value <0.05 was considered significant. Analysis was performed using the R-package multtest [35]. Genes in GISTIC regions were identified by matching GISTIC coordinates to the hg17 RefGene file obtained from the dChip software website.

# Construction of a HER2– breast cancer reference data sets

A 554-sample HER2– reference breast cancer data, excluding *HER2*-amplified tumors, was constructed from four independent breast cancer data sets. The Chin et al. [31] data set (n=145) was segmented using CBS [27] (α=0.01) after BAC probe positions had been updated to the UCSC Human Genome browser build 17. *HER2*-amplified tumors were identified by IHC annotations [31] (n=17) and removed. Regions of genomic gain and loss were identified by applying fixed log_2_ratio thresholds (±0.1) to CBS data.

The Fridlyand et al. [32] data (n=67) was segmented using CBS (α=0.01) after BAC probe positions had been updated to the UCSC Human Genome browser build 17. *HER2*-amplified tumors (n=16) were detected by BAC clone DMPC-HFF#1-61H8 with log_2_ratio >0.5 and removed. Regions of genomic gain and loss were identified by applying fixed log_2_ratio thresholds (±0.1) to CBS data.

The Adelaide et al. [33] data set (n=93) was obtained as segmented data. *HER2*-amplified tumors (n=5) were identified by the average log_2_ratio of oligonucleotide probes matching *HER2* >0.5, and removed. Regions of genomic gain and loss were identified by applying fixed log_2_ratio thresholds (±0.15) to segmented data. In addition, RMA normalized gene expression profiles for 74 tumors were obtained from [33] and log2 transformed. Each probe was mean centered across the entire gene expression data set. Molecular subtype classification was performed by correlation to gene expression centroids reported by Hu et al. [41]. Probes were matched based on gene identity to the 306 genes in the Hu et al. centroids. Subtypes were assigned to samples based on highest Pearson correlation to a subtype centroid. A correlation cut-off of 0.2 was used as threshold and samples with the highest correlation <0.2 were set as unclassified.

The Jönsson et al. (Jönsson et al., submitted 2009) data set (n=359) was processed similarly as the 200 HER2+ tumors, using the same BAC aCGH platform, CBS analysis and calling of copy number aberrations. *HER2*-amplified tumors (n=72) were identified by the average log_2_ratio of five BAC clones mapping to HER2 (RP11-94L15, RP11-62N23, RP11-563O4, RP11-689B15, RP11-610O22) >0.5 and removed. All data sets were next transformed to a common 100kb probe set as described [34] and merged.

Threshold for amplification was set to segmented log_2_ratio ≥0.5, and for high-level amplification to segmented log_2_ratio >0.8 for HER2– tumors in the reference data set. A lower threshold for high-level amplifications, compared to HER2+ tumors, were chosen due to different characteristics (aCGH platforms) for individual data sets included in the combined reference data set.

# Gene expression analysis

Gene expression data for 87 of the 200 *HER2*-amplified tumors were available as either oligonucleotide data (n=58) from the Jönsson et al. data set or as cDNA data (n=29) [22] part of larger BC data sets (n=359 and n=168 respectively). Data sets were individually processed as described (Jönsson et al. submitted, and [22]). Both data sets were classified according to the gene expression subtypes [15], using the Hu et al. [41] gene list.
