# Supplementary material for: High-resolution genomic and expression analyses of copy number alterations in HER2-amplified breast cancer
Source: Breast Cancer Res. 2010 May 6;12(3):R25. doi: 10.1186/bcr2568 (PMC2917012; doi:10.1186/bcr2568)
Supplement: Additional file 3 — Patient and tumor characteristics for HER2-negative tumors. A Word document containing a table of clinical data for HER2- tumors in the reference breast cancer data set. [file bcr2568-S3.DOC]

Additional File 3. Tumor characteristics for the 554-sample HER2– breast cancer data set.

|  | Chin et al. | Fridlyand et al. | Adelaide et al. | Jönsson  et al. |
| --- | --- | --- | --- | --- |
| Number of tumors | 128 | 51 | 88 | 287 |
| Estrogen receptor-status |  |  |  |  |
| Positive | 87 | 22 | NA* | 197 |
| Negative | 41 | 29 | NA | 79 |
| Histological grade |  |  |  |  |
| Grade 1 | 16 | NA | NA | 25 |
| Grade 2 | 52 | NA | NA | 82 |
| Grade 3 | 57 | NA | NA | 77 |
| Lymph node-status |  |  |  |  |
| Negative | 63 | 17 | NA | 164 |
| Positive | 65 | 29 | NA | 100 |
| Tumor size |  |  |  |  |
| ≤20mm | 65 | 17 | NA | 123 |
| >20mm | 60 | 34 | NA | 132 |
| Mean size mm (SD) | 24 (12) | 34 (18) | NA | 25 (14) |
| Gene expression subtypes |  |  |  |  |
| Number of basal-like | 24 | NA | 41 | 70 |
| Number of luminal A | 41 | NA | 23 | 89 |
| Number of luminal B | 10 | NA | 6 | 61 |
| Number of normal-like | 10 | NA | 0 | 25 |
| Age |  |  |  |  |
| Median age in years (range) | 53 (28-88) | 70 (29-94) | NA | 50 (28-88) |
| Overall survival |  |  |  |  |
| Number of deaths | 46 | 24 | NA | 120 |
| Within 5 years | 30 | 19 | NA | 58 |
| Median survival in years | 7.4 | 5.0 | NA | 8.5 |
| Median survival in years for patients still alive | 9.0 | 6.8 | NA | 12.9 |

* Data not available.
