# Supplementary material for: High-resolution genomic and expression analyses of copy number alterations in HER2-amplified breast cancer
Source: Breast Cancer Res. 2010 May 6;12(3):R25. doi: 10.1186/bcr2568 (PMC2917012; doi:10.1186/bcr2568)
Supplement: Additional file 4 — HER2 copy number evaluation and TOP2A mRNA expression levels. A pdf file containing figures of the result of the comparison of HER2 copy number estimates between aCGH and FISH for 13 FFPE samples (S1A) and mRNA expression levels and S-phase fractions for HER2+ and HER2- tumors in the Jönsson et al. data set (S1B). [file bcr2568-S4.PDF]

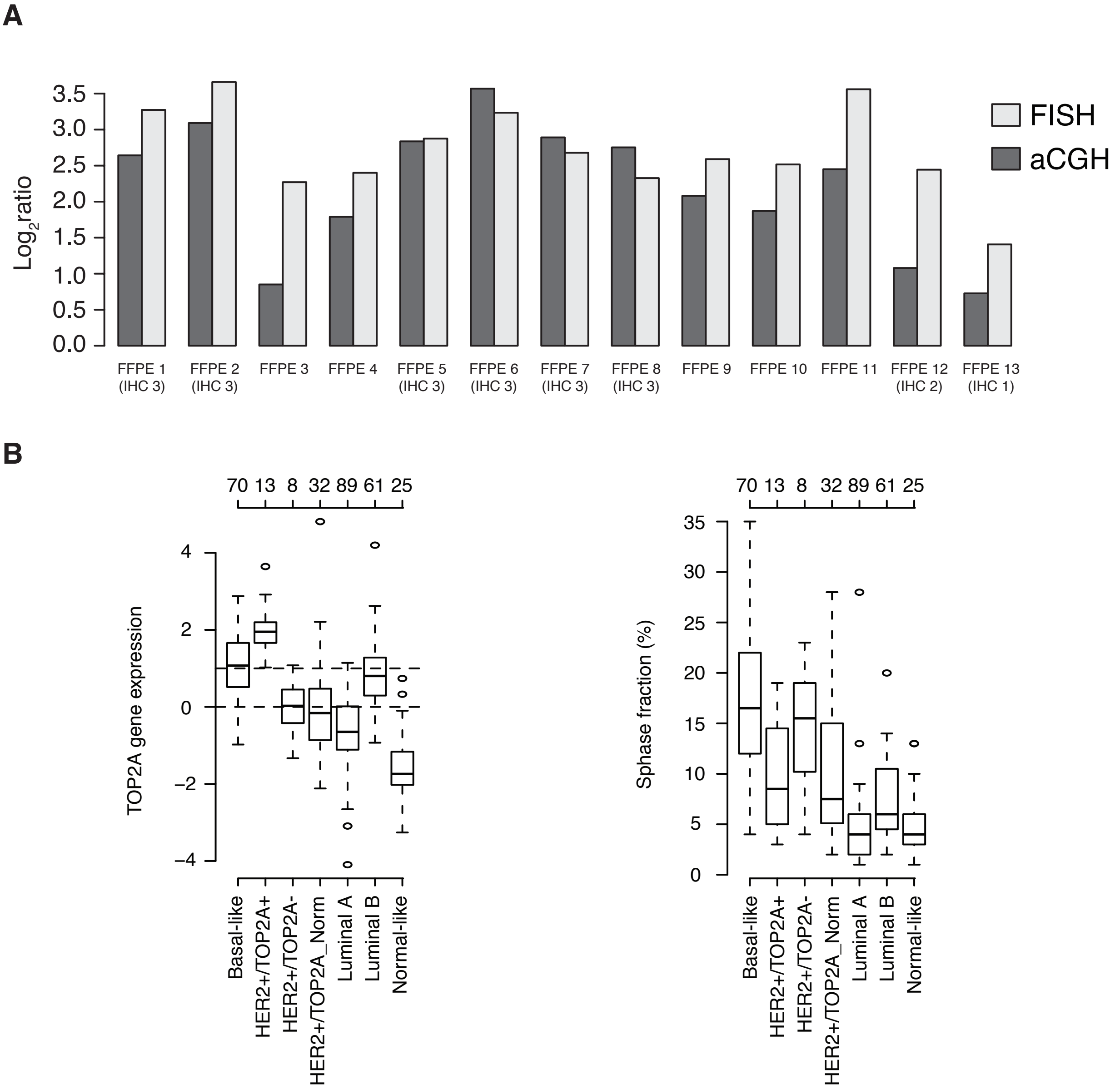

Supplementary Figure S1. *HER2* estimates by FISH and aCGH, and *TOP2A* gene expression levels in subtypes of breast cancer. (**A**) Comparison of log$_2$ratios obtained by FISH and aCGH for *HER2* from 13 FFPE HER2+ breast tumors. Values within parentheses indicate IHC score for tumors. For aCGH, estimates were calculated as the average log$_2$ratio of seven BAC clones covering *HER2* (RP11-94L15, RP11-62N23, RP11-563O4, RP11-689B15, RP11-610O22, RP11-909L6 and RP11-1065L22) after normalization. FISH ratios were calculated as *HER2* red signals divided by chromosome 17 green signals in 20 nuclei and subsequently log2-transformed. (**B**) mRNA expression levels for *TOP2A* (left) and S-phase fractions (right) for HER2+ tumors part of the complete Jönsson et al. data set stratified by TOP2A status, and for HER2– tumors in the Jönsson et al. data set classified according to gene expression subtypes. Numbers above boxes indicate group sizes.
