# Supplementary material for: High-resolution genomic and expression analyses of copy number alterations in HER2-amplified breast cancer
Source: Breast Cancer Res. 2010 May 6;12(3):R25. doi: 10.1186/bcr2568 (PMC2917012; doi:10.1186/bcr2568)
Supplement: Additional file 6 — Recurrent high-level amplifications in HER2-amplified breast cancer. A Word file describing recurrent high-level amplifications, excluding chromosome 17, in HER2+ breast tumors including genes in amplicons significantly correlated between mRNA expression levels and copy numbers. [file bcr2568-S6.DOC]

Additional File 6. Shortest region of overlap of recurrent amplifications in HER2+ breast cancer, excluding chromosome 17.

| Region^a^ | All^b^ | ER–^b^ | ER+^b^ | Genes and miRNAs^c^ |
| --- | --- | --- | --- | --- |
| 1q21.2  chr1:147248800-147699345 | 5 | 4 | 1 | *RPRD2*,* ***TARSL1****,* ***ECM1****, TSRC1*, MCL1,* ***ENSA****,* ***GOLPH3L****, HORMAD1*, CTSS, CTSK, ARNT* |
| 1q32.1-1q32.2  chr1:201991026-203691211 | 7 | 2 | 5 | *NUAK2, BCA3*, KLHDC8A*, LEMD1*, PCTK3, ELK4, SLC45A3*, NUCKS1, RAB7L1,* ***SLC41A1****, PM20D1*, SLC26A9*, AVPR1B*, CTSE, SRGAP2, IKBKE, RASSF5,* ***LGTN****,* ***DYRK3****, MAPKAPK2, IL10*, IL19, IL20*, IL24, FAIM3, PIGR, FCAMR*, YOD1,* ***PFKFB2****, C4BPB, C4BPA*, mir-135b* |
| 5p15.33  chr5:431268-1062674 | 7 | 4 | 2 | *AHRR*, SEC6L1*, SLC9A3, Cep72, TPPP*, ZDHHC11*, BRD9, TRIP13, NKD2* |
| 6p23  chr6:14071912-15252036 | 6 | 4 | 2 | RNF182*, CD83 |
| 6q21  chr6:107334854-107624768 | 9 | 6 | 2 | C6orf203*, PDSS2, mir-587 |
| 8p12  chr8:37418779-38488995 | 6 | 2 | 4 | ***ZNF703****, SPFH2, PROSC, GPR124, BRF2*,* ***RAB11FIP1****, GOT1L1*, ADRB3, EIF4EBP1,* ***ASH2L****,* ***STAR****,* ***LSM1****,* ***BAG4****,* ***DDHD2****,* ***PPAPDC1B****, WHSC1L1, LETM2*,* ***FGFR1*** |
| 8p11.21  chr8:41795492-42450341 | 7 | 3 | 4 | ***MYST3, AP3M2****, PLAT,* ***IKBKB****,* ***POLB****, DKK4*,* ***VDAC3****, SLC20A2* |
| 8q21.2-q21.3  chr8:86620595-87047134 | 13 | 7 | 6 | REXO1L1* |
| 8q22.3  chr8:102322735-103438234 | 13 | 8 | 5 | *GRHL2, EDD*, NCALD, RRM2B** |
| 8q23.3-q24.11  chr8:117287112-118299631 | 17 | 10 | 7 | ***EIF3S3****, C8orf53*, RAD21*, SLC30A8** |
| 8q24.21  chr8:128784030-129096105 | 23 | 11 | 12 | MYC, mir-1204, mir-1205, mir-1206 |
| 11q13.3  chr11:69132863-69647936 | 9 | 1 | 8 | ***CCND1****, ORAOV1*, FGF19, FGF4*, FGF3*,* ***TMEM16A*** |
| 11q13.5  chr11:76260080-76998741 | 8 | 2 | 6 | ***PHCA****, B3GNT6*, CAPN5, OMP*,* ***MYO7A****, GDPD4*,* ***PAK1****, AQP11* |
| 19q13.42  chr19:60725260-60850444 | 9 | 2 | 7 | *ZNF579, FIZ1*,* ***ZNF524****, ZNF580, ZNF581* |
| 20q13.2  chr20:51487854-51915327 | 10 | 4 | 5 | *ZNF218*,* ***ZNF217*** |
| 20q13.32  chr20:55564230-55814971 | 10 | 7 | 2 | *PCK1, ZBP1*, TMEPAI* |

^a^ Region is defined as the shortest region of amplification overlap (SRO). Genomic positions given for hg17.

^b^ Number of HER2+ tumors with high-level amplification.

^c^ Genes or miRNA in SRO. Genes in bold significantly correlated between aCGH and matched gene expression data. miRNAs were identified by conversion of hg17 positions to hg18 followed by matching to miRBase ver 13.0.

* Gene expression data not available.
