# Supplementary material for: High-resolution genomic and expression analyses of copy number alterations in HER2-amplified breast cancer
Source: Breast Cancer Res. 2010 May 6;12(3):R25. doi: 10.1186/bcr2568 (PMC2917012; doi:10.1186/bcr2568)
Supplement: Additional file 8 — Expression of ER gene expression modules in HER2-amplified and HER2-negative breast cancer. A pdf file containing two subpanels illustrating: (1) differences in expression of two ER gene expression modules in the Jönsson et al. data set for HER2+ tumors stratified according to ER status, and (2) HER2- tumors classified according to gene expression subtypes. [file bcr2568-S8.PDF]

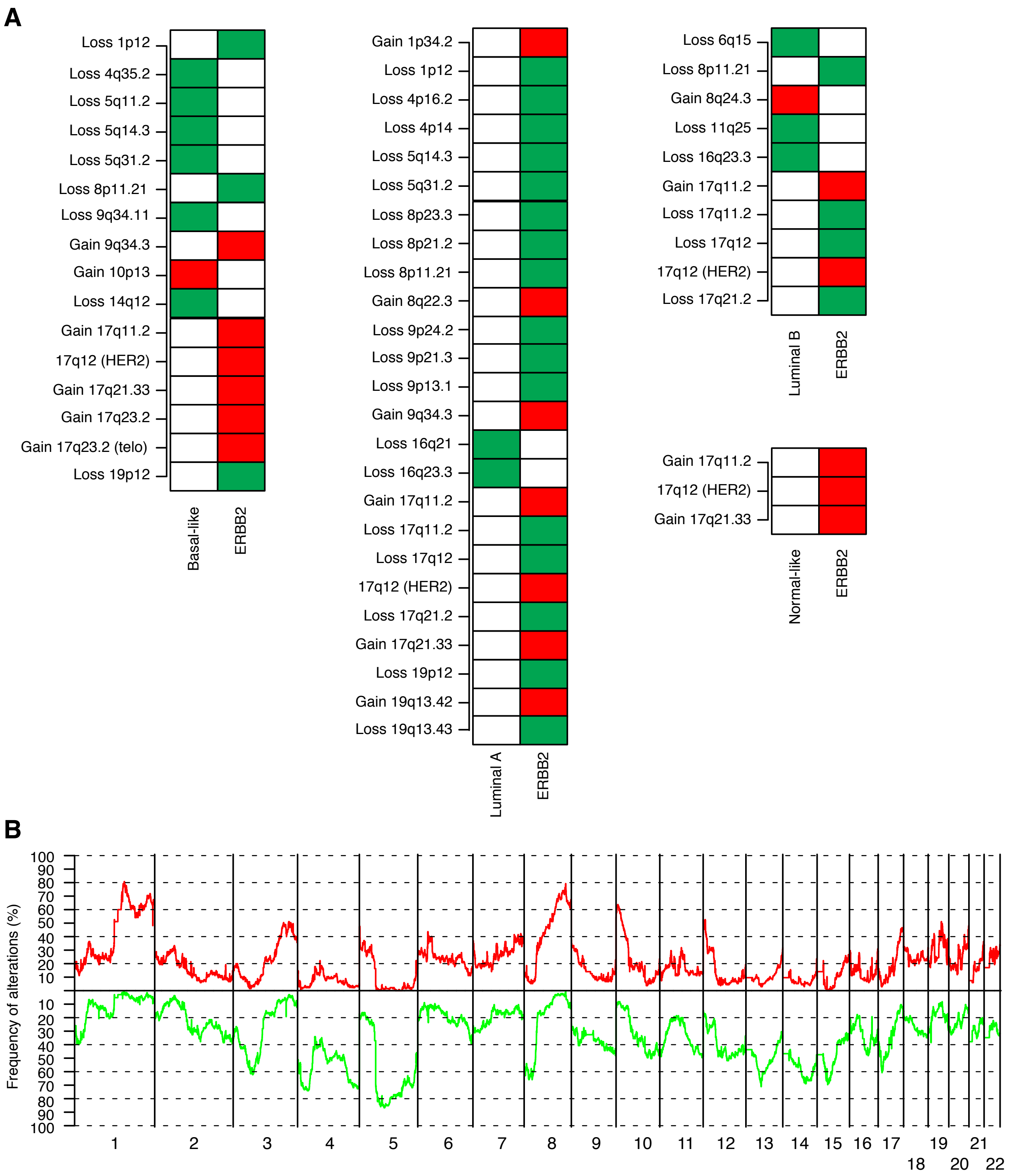

Frequency of CNAs in ERBB2 subtype classified HER2+ tumors in relation to HER2– tumors classified according to molecular subtypes. (**A**) Discriminating GISTIC regions between ERBB2 subtype classified HER2+ tumors and HER2– tumors classified as basal-like, luminal A, luminal B or normal-like. GISTIC regions identified by Bonferroni adjusted Fisher's exact test ($p < 0.05$). Each box represents a GISTIC region. Red boxes indicates more frequent gain, green boxes indicates more frequent loss. (**B**) Frequency of CNAs in HER2- basal-like tumors (n = 135). Frequency of gain is shown in red and loss in green.
