# Supplementary material for: High-resolution genomic and expression analyses of copy number alterations in HER2-amplified breast cancer
Source: Breast Cancer Res. 2010 May 6;12(3):R25. doi: 10.1186/bcr2568 (PMC2917012; doi:10.1186/bcr2568)
Supplement: Additional file 9 — Frequency of GISTIC regions in HER2-amplified and HER2-negative breast cancer according to gene expression subtypes. A pdf file containing two panels illustrating: (1) GISTIC regions significantly different between HER2+ tumors classified to the ERBB2 gene expression subtype, compared to HER2- tumors classified as basal-like, luminal A, luminal B and normal-like subtype, and (2) CNA frequency in HER2- basal-like classified tumors. [file bcr2568-S9.PDF]

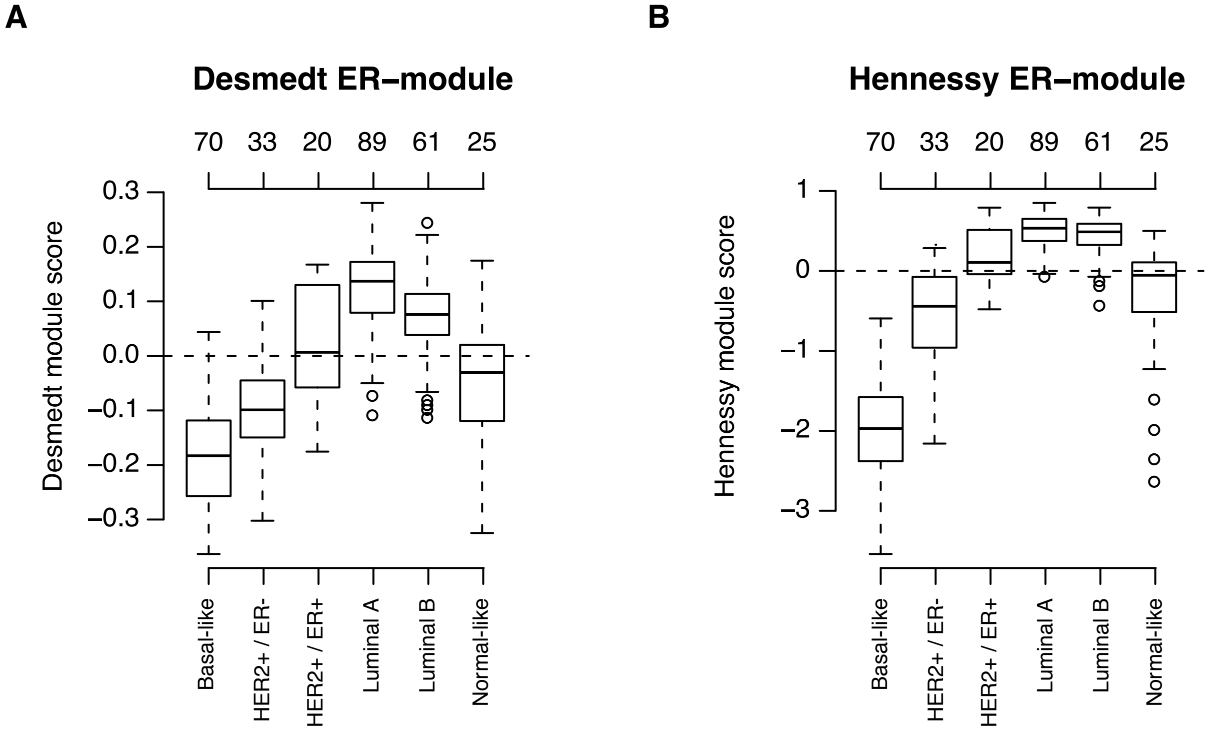

Differences in expression of two ER gene expression modules in the Jönsson et al. data set for HER2+ tumors stratified by ER status, and HER2– tumors classified according to gene expression subtypes. Top-axis indicates number of tumors in each subgroup. Gene expression log_2ratios were ranked for each sample. A rank sum was next calculated for each sample and module based on overlapping genes. The module score for an individual sample was defined as the rank sum for the sample divided by average ranksum across all samples and log2-transformed. (**A**) Module scores for the Desmedt et al. [42] ER gene module. (**B**) Module scores for the Hennessy et al. [43] ER gene module described in the article supplemental data.
