# Supplementary material for: High-resolution genomic and expression analyses of copy number alterations in HER2-amplified breast cancer
Source: Breast Cancer Res. 2010 May 6;12(3):R25. doi: 10.1186/bcr2568 (PMC2917012; doi:10.1186/bcr2568)
Supplement: Additional file 10 — Copy number alterations in HER2-amplified breast cancer stratified by ER status. A pdf file containing two subpanels illustrating CNA frequencies in HER2+/ER- tumors and HER2+/ER+ tumors, respectively. [file bcr2568-S10.PDF]

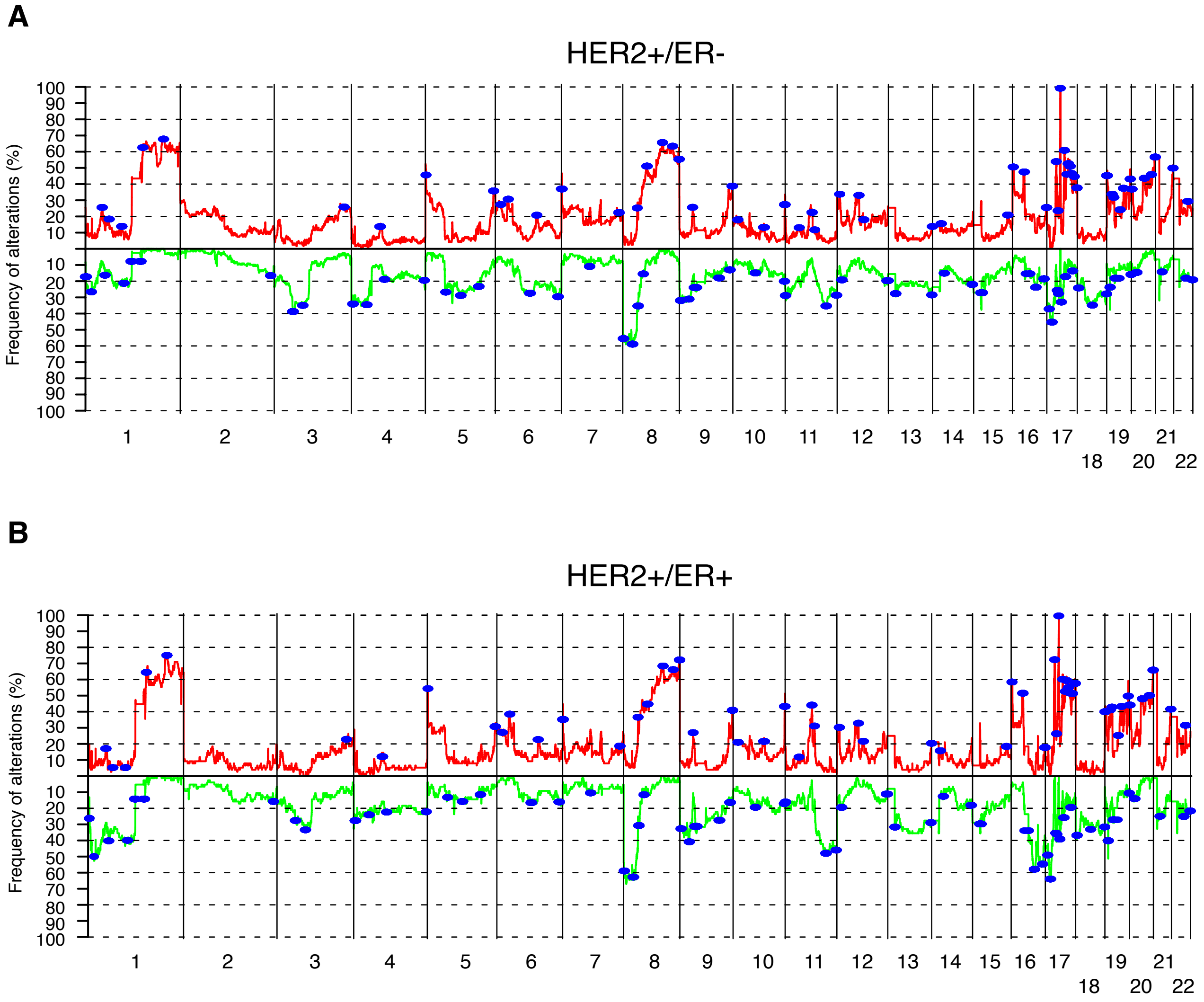

Frequency of CNAs in HER2+ breast tumors stratified by ER status. Frequency of gain is shown in red and loss in green. Blue regions indicate significant CNAs identified by GISTIC-analysis using all 200 tumors. (**A**) HER2+/ER– tumors (n = 122). (**B**) HER2+/ER+ tumors (n = 76).
